# Lipidomic signatures of aortic media from patients with atherosclerotic and nonatherosclerotic aneurysms

**DOI:** 10.1038/s41598-019-51885-4

**Published:** 2019-10-29

**Authors:** Kosuke Saito, Hiroaki Yagi, Keiko Maekawa, Mitsuhiro Nishigori, Masaki Ishikawa, Sayaka Muto, Tsukasa Osaki, Yutaka Iba, Kenji Minatoya, Yoshihiko Ikeda, Hatsue Ishibashi-Ueda, Hitoshi Ogino, Hiroaki Sasaki, Hitoshi Matsuda, Yoshiro Saito, Naoto Minamino

**Affiliations:** 1 Division of Medical Safety Science, National Institute of Health Sciences, Kanagawa, Japan; 2 Department of Molecular Pharmacology, National Cerebral and Cardiovascular Center Research Institute, Osaka, Japan; 3 Department of Pathology, National Cerebral and Cardiovascular Center, Osaka, Japan; 4 Department of Vascular Surgery, National Cerebral and Cardiovascular Center, Osaka, Japan; 5 Present Address: Omics Research Center, National Cerebral and Cardiovascular Center, Osaka, Japan

**Keywords:** Aortic diseases, Aneurysm

## Abstract

Aortic aneurysms are associated with fatal aortic rupture. Current therapeutic approaches are limited to implantation of aortic prostheses and stent-grafts; no effective drugs are available because the pathogenic mechanisms of aortic aneurysms remain unclear. Here, we aimed to elucidate the molecular mechanisms of the initiation and progression of aortic aneurysm by lipidomics. We performed lipidomics analyses of lipids in the aortic media of normal, border, and aneurysm areas from patients with thoracic atherosclerotic aortic aneurysm (N = 30), thoracic nonatherosclerotic aortic aneurysm (N = 19), and abdominal atherosclerotic aortic aneurysm (N = 11) and from controls (N = 8) using liquid chromatography and mass spectrometry. Significant alterations were observed in the lipid profiles of patients with atherosclerotic aortic aneurysms and to a lesser extent in those with nonatherosclerotic aneurysms. Increased triacylglycerols (TGs) and decreased ether-type phosphatidylethanolamines (ePEs) were observed throughout the normal, border, and aneurysm areas of thoracic and abdominal atherosclerotic aortic aneurysms. Prostaglandin D_2_ increased, but ePEs and TGs decreased in normal areas of thoracic atherosclerotic aortic aneurysms and thoracic nonatherosclerotic aortic aneurysms compared with the control tissues. These findings expand our knowledge of metabolic changes in aortic aneurysms and provide insights into the pathophysiology of aortic aneurysms.

## Introduction

Aortic aneurysm is a progressive disease with aortic dilation, mainly caused by remodelling of the aortic wall by atherosclerosis; morbidities associated with aortic aneurysm are increasing worldwide owing to lifestyle changes. Importantly, aortic aneurysm can lead to fatal consequences of aortic dissection and rupture^1–3^. Most aortic aneurysms are asymptomatic until rupture, and most patients with aortic aneurysms are diagnosed when undergoing diagnostic imaging for other suspected diseases or during voluntary medical examinations. Moreover, current therapeutic approaches are limited to implantation of aortic prostheses and stent-grafts, and no effective drugs have been developed. Therefore, identifying novel diagnostic biomarkers and appropriate molecular targets for drug development is essential. However, the molecular mechanisms initiating, promoting, and exacerbating aortic aneurysms are still poorly understood, although inflammation, oxidative stress, matrix degradation, thrombosis, and hemodynamic forces contribute to these processes^4,5^.

The aortic wall contains a concentric medial layer, the aortic media, which is made up of a relatively uniform lamellar tissue layer composed of vascular smooth muscle cells and extracellular matrices, such as elastin and collagen; this layer is sandwiched by intima and adventitia in the aortic wall and separated from the blood stream^1,6,7^. The aortic media in the aortic wall is the focus of atherosclerotic remodelling; this tissue can be collected from the normal-to-aneurysm areas of surgically excised aortic aneurysm tissue, even if it has thinned in the advanced stages of the disease. To identify target molecules for the development of therapies and drugs, the aortic media of aortic aneurysms is a promising target tissue and can be subjected to Omics analyses to elucidate the pathophysiology of atherosclerotic aortic aneurysms.

Lipids, such as phosphoglycerolipids, sphingolipids, and neutral lipids, play key roles in a variety of physiological and biological processes as membrane constituents, energy sources, and second messengers^8–10^. In addition, another class of lipids oxylipins, including prostaglandins and leukotrienes, play important roles in inflammation^11^. Therefore, profiling of the lipid status could provide novel mechanistic insights into the pathophysiology of diseases. Recently, a liquid chromatography/mass spectrometry (LC/MS)-based technique, called lipidomics, which simultaneously monitors alterations in a broad range of lipids^12,13^, was used to screen changes in lipid statuses in several diseases, address the pathophysiology of the diseases, and/or identify biomarkers. For example, phosphatidylethanolamine (PE) levels were found to be dramatically decreased in clear cell renal cell carcinoma by lipidomics, whilst consolidated analysis with transcriptome and cell based analysis showed that the PE synthetic pathway was suppressed in this form of carcinoma^14^. In addition, an increasing number of studies performing lipidomic analyses have demonstrated dysregulated lipid metabolism, such as excess glycosphingolipid levels, in the plasma and brain tissue of patients with Parkinson’s disease^15^. These lipidomics studies have highlighted the possibility that abnormal lipid composition due to dysregulated lipid metabolism is one of the main causes of the pathogenesis of these diseases. The major cause of aortic aneurysm is atherosclerosis, wherein pathological lipid accumulation typically results in atheroma formation and induces the remodelling of the aortic wall, predominantly in the aortic media. Thus, lipidomics of the aortic media of aortic aneurysms could also help to elucidate the fundamental lipid status of this fatal disease, leading to mechanistic insights into the pathophysiology of aortic aneurysms.

In the present study, we collected aortic media from patients with thoracic atherosclerotic aortic aneurysm (TAAA), thoracic nonatherosclerotic aortic aneurysm (TNAA), and abdominal atherosclerotic aortic aneurysm (AAAA) and used these samples to determine the lipidomic signatures of these conditions. For TAAA and TNAA, we also collected thoracic aorta from patients without any vascular disease as controls. Our results provided fundamental information for elucidating the molecular mechanisms of atherosclerotic aortic aneurysms and identifying potential drug targets for the treatment of this disease.

## Results

### Patient characteristics

Patients with TAAA and AAAA showed no differences in age; however, patients with TAAA and AAAA were older than the control patients with control normal thoracic aorta (CNTA) (*p* < 0.001 for TAAA, *p* = 0.004 for AAAA; Table 1), since all the control patients without vascular disease had received age-restricted heart transplants. Patients with TNAA were younger than patients with TAAA (*p* = 0.005) mainly because majority of the TNAA group had hereditary disorders. In all groups of aortic aneurysms, men were predominant (82%). The average maximum aneurysmal diameters of TAAA, TNAA, and AAAA, were 56.5 ± 1.2, 54.5 ± 1.5, and 57.0 ± 3.1 mm, respectively, and no differences were observed among their diameters. The differences between the greatest dimensions and the anastomotic site diameters of TAAA and TNAA were almost the same, indicating similarities in the dimensions of these tissues. In contrast, AAAA (32.6 ± 2.5 mm) showed significantly greater differences between maximal aneurysmal and anastomotic site diameters than either TAAA (21.1 ± 1.4 mm, *p* = 0.001) or TNAA (21.8 ± 1.7 mm, *p* = 0.012) (Table 1).

**Table 1:** Pathophysiological characteristics of patients with aortic aneurysm and non-vascular disease controls enrolled in this study.

		TAAA	TNAA	AAAA	CNTA
No. of patients		30	19	11	8
Age (years)		72.0 ± 1.3*	57.3 ± 3.5^#^	67.9 ± 2.2*	28.0 ± 4.2
Sex (male/female)		28/2	13/6	8/3	7/1
Body mass index (kg/m^2^)		23.5 ± 0.5	23.5 ± 0.9	23.7 ± 0.8	20.7 ± 1.1
Aneurysm size^a^		21.1 ± 1.4	21.8 ± 1.7^$^	32.6 ± 2.5^#^	—
Complication					
Hypertension (+/−)		26/4	13/6	7/4	1/7
Diabetes mellitus (+/−)		5/25	2/17	1/10	0/8
Hyperlipidemia (+/−)		17/13	2/17	3/8	0/8
Statin treatment (+/−)		16/14	1/18	3/8	0/8
Biochemical test					
Total cholesterol (mg/dL)		171 ± 6	211 ± 6*^#^	190 ± 12*	144 ± 7
Oxidised low-density lipoprotein (mg/dL)		130 ± 7	144 ± 10	131 ± 16	—
High-density lipoprotein cholesterol (mg/dL)		47.5 ± 1.9	53.2 ± 2.8	43.6 ± 3.7	47.0 ± 3.5
Low-density lipoprotein cholesterol (mg/dL)		102 ± 5	127 ± 6*^#^	113 ± 10	80 ± 6
C-reactive protein (mg/dL)		0.20 ± 0.05	0.13 ± 0.04*	0.52 ± 0.32	1.09 ± 0.32
Creatinine (mg/dL)		1.36 ± 0.33	0.79 ± 0.03^#^	1.43 ± 0.48	0.85 ± 0.09
Pathological examination					
Atherosclerosis^b^	NOR	0	3	0	3
	IL	0	13	0	2
	AT	3	3	0	0
	CL	27	0	11	0
	UK	0	0	0	3
Medionecrosis^c^	NOR	1	0	0	0
	M	0	3	0	5
	IM	13	8	0	0
	S	16	8	11	0
	UK	0	0	0	3
Aortic elastic fibre fragmentation^c^	NOR	0	1	0	3
	M	2	4	0	2
	IM	11	3	0	0
	S	17	11	11	0
	UK	0	0	0	3
Cystic medial necrosis^c^	NOR	29	9	11	5
	M	1	3	0	0
	IM	0	3	0	0
	S	0	4	0	0
	UK	0	0	0	3

Data are means ± SEM. Statistical significance (p < 0.05) was observed: *compared with CNTA, ^#^compared with TAAA, and ^$^compared with AAAA.

^a^Aneurysm size indicates difference (mm) between greatest dimension and anastomotic site diameter.

^b^Atherosclerosis is classified into 4 classes (normal, NOR; initial lesion, IL; atheroma, AT; complicated lesion, CL) and unknown (UK).

^c^Medionecrosis, aortic elastic fibre fragmentation, and cystic medial necrosis are classified into 4 classes (normal, NOR; mild, M; intermediate, IM, and severe, S) and unknown (UK).

When analysing underlying diseases as risk factors of atherosclerosis, hypertension was commonly and dominantly observed in the three groups of aortic aneurysms (77%), and diabetes mellitus was present in less than 20% of patients in these groups. Hyperlipidaemia was more frequently observed in the TAAA group (57%). Nearly all patients with TAAA and AAAA who were diagnosed as having hyperlipidaemia received statins. In contrast, patients in the control group had no underlying diseases, except for one patient who had hypertension. In blood lipid tests, oxidised low-density lipoprotein levels did not differ among the three aortic aneurysm groups, and high-density lipoprotein levels did not differ among the aortic aneurysm groups and the control group. The TNAA group showed higher total cholesterol levels than the control (*p* < 0.001) and TAAA groups (*p* = 0.001), and higher low-density lipoprotein levels than the TAAA group (*p* = 0.018) and the control group (*p* = 0.001). The AAAA group showed higher total cholesterol levels than the control group (*p* = 0.031). Although some data, including the higher total cholesterol and low-density lipoprotein levels in the TNAA group, were unexpected, the high ratio of statin prescriptions to patients with hyperlipidaemia in the TAAA and AAAA groups may have reduced these two indices. C-reactive protein and creatinine levels were not dramatically different among the three aortic aneurysm groups.

In pathological examinations, more than 90% of TAAA and AAAA cases were classified as complicated atherosclerotic lesions of the most advanced stage, and the remaining less than 10% were considered atheroma; in contrast, 16% and 68% of TNAA cases were classified as atheromas and initial lesions, respectively. In medionecrosis and aortic elastic fibre fragmentation, TAAA and AAAA showed more severe tendencies than TNAA. In the cystic medial necrosis, more advanced phenotypes were observed in TNAA than in TAAA and AAAA.

### Histology

**Figure 1:**
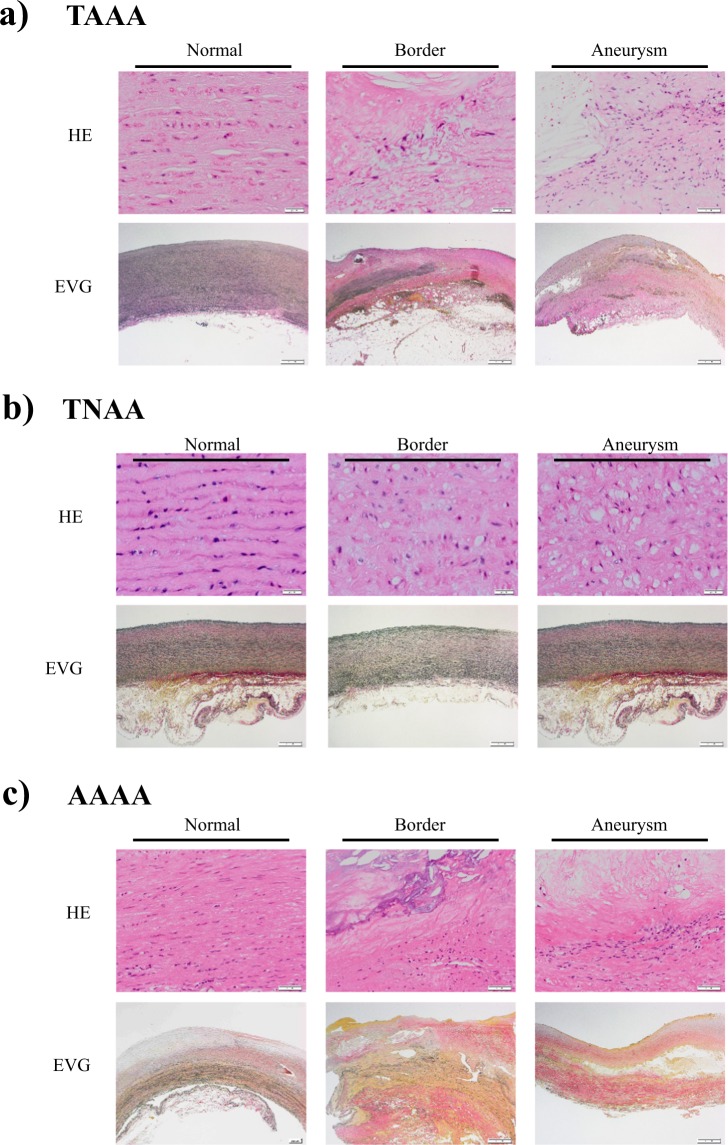
Histological features of aortic walls of thoracic atherosclerotic aortic aneurysm (TAAA), thoracic nonatherosclerotic aortic aneurysm (TNAA), and abdominal atherosclerotic aortic aneurysm (AAAA). (**a–c**) Aortic wall staining results for TAAA, TNAA, and AAAA, respectively. Left, middle, and right panels are data for normal, border, and aneurysm areas, respectively. Upper and lower panels in A–C are staining data for hematoxylin and eosin (HE) with higher magnification and elastica van Gieson (EVG).

As shown in Fig. 1a,c, diffuse intimal thickening without disruption of the medial architecture (left normal panels) in nondilated aortic walls was found to be a precursor lesion for advanced lipid plaques with calcification, fibro-inflammatory lesions, and disruption of medial architecture, in transitional (mid border panels) and dilated aortic walls (right aneurysm panels) in TAAA (Fig. 1a) and AAAA (Fig. 1c) groups. Histopathological findings suggested that lipids or products of lipid oxidation were present in advanced aortic aneurysms with atherosclerosis in these two groups. In contrast, the aortic walls in the TNAA group (Fig. 1b) showed nonatherosclerotic lesions with smooth surfaces in the lumen. Histological characteristic features in the TNAA group shown in Fig. 1 included more diffuse medial degeneration with loss of smooth muscle cells and more collagen deposition (Fig. 1b, middle panels) than control samples (Fig. 1b, left panels). Medial degeneration that developed in dilated aortic walls of the TNAA group (Fig. 1b, right panels) was associated with more vacuolar smooth muscle cells than nondilated and transitional aortic walls in the TAAA and AAAA groups (Fig. 1a,c, left and middle panels, respectively).

### Lipid molecules detected and quantified in the aortic media

**Figure 2:**
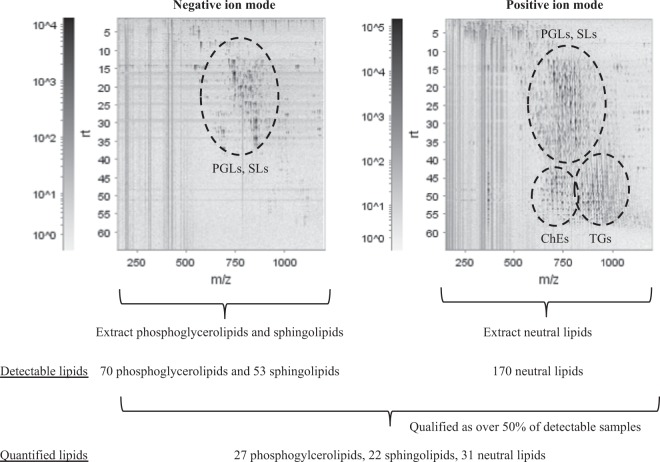
Representative two-dimensional map of LC/MS nontargeted lipidomic analysis using aortic media with TAAA, TNAA, and AAAA. Phosphoglycerolipids and sphingolipids were detected in negative ion mode, and neutral lipids were detected in positive ion mode, yielding 70 phosphoglycerolipids, 53 sphingolipids, and 170 neutral lipids. In addition, 27 phosphoglycerolipids, 22 sphingolipids, and 31 neutral lipids were qualified as quantified lipids. PGLs: phosphoglycerolipids, SLs: sphingolipids, ChEs: cholesterolesters, TGs: triacylglycerols, *m/z*: mass-to-charge ratio.

Nontargeted lipidomic analysis was performed to detect and determine the levels of phosphoglycerolipids, sphingolipids, and neutral lipids in various aortic media samples (Fig. 2). By extracting ion peaks, the nontargeted lipidomic analysis yielded 293 detectable lipid molecules (70 phosphoglycerolipids, 53 sphingolipids, and 170 neutral lipids; Tables 2 and S1). Consistent with previous observations showing that oxidised cholesterolesters (ChEs) were elevated in atherosclerotic lesions^16,17^, five oxidised cholesterolesters, including ChE(18:2) + 2O, were detected at a higher frequency in the border and aneurysm areas of TAAA and AAAA than in the normal areas of TAAA and AAAA and all areas of TNAA (Supplemental Fig. S1). Subsequently, lipid molecules detectable (exceeding the data cut off point) in more than half of samples were selected for comparison of lipid profiles. In total, 80 lipid molecules (27 phosphoglycerolipids, 22 sphingolipids, and 31 neutral lipids; Supplemental Tables S2 and S3) were found to be over the quantifiable threshold and were subjected to subsequent analyses.

**Table 2:** Identified lipid classes and numbers of individual lipid molecules by nontargeted LC/MS in aortic media.

Lipid type	Lipid classes	Number of molecules (oxidized)
Phospholipid	lysophosphatidylcholine (LPC)	4
	phosphatidylcholine (PC)	24
	ether-type PC (ePC)	9
	phosphatidylethanolamine (PE)	11
	ether-type PE (ePE)	15
	phsophatidylinositol (PI)	6
	phosphatidylglycerol/bismonophosphotidate (PG/BMP)	1
Sphingolipid	sphingomyelin (SM)	31 (2)
	ceramide (Cer)	8
	glycosylceramide (GCer)	14
Neutral lipid	diacylglycerol (DG)	7
	cholesterol/cholesterolester (Ch/ChE)	43 (5)
	coenzyme Q (CoQ)	1
	triacylglycerol (TG)	114
	ether-type TG (eTG)	5
	total	293

### Comparison of nontargeted lipid profiles among normal, border, and aneurysm areas in TAAA and TNAA

**Figure 3:**
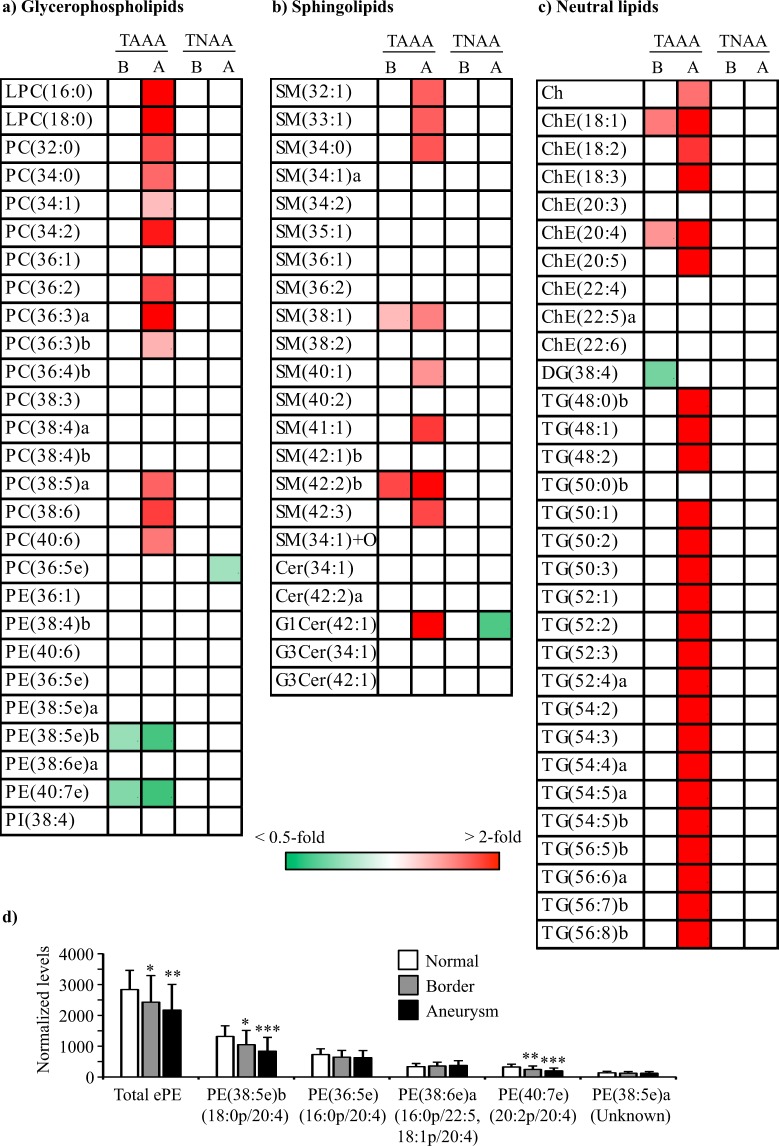
Heatmaps for molecules identified using nontargeted lipidomic analysis of aortic media in normal, border, and aneurysm areas of TAAA and TNAA. (**a–c**). Heatmaps with significantly different (*p* < 0.05) lipid molecules were generated using mean fold changes in the levels of individual molecules calculated as ratios of each area to the normal area. Vacant white cells indicate molecules that were quantified, but their levels were not significantly different. (**d**) Levels of ether-type PEs of the three areas from TAAA. Data are presented as summed normalised ion peak heights of each peak of the sample groups and shown as means ± SDs. *p < 0.05, **p < 0.01, ***p < 0.001 in paired t-tests comparing border or aneurysm areas and normal areas. Combinations of fatty alcohol and fatty acid are shown underneath each molecule. B: border, A: aneurysm. The last lowercase letters (a,b) in lipid names represent molecules with the same carbon and double bond numbers, but have different fatty acid compositions (molecules with same m/z in MS with different retention time in LC). Other abbreviations are described in Table 2.

First, we compared nontargeted lipid profiles of aortic media among normal, border, and aneurysm areas obtained from patients with TAAA and TNAA (Fig. 3a–c). Seven and 48 of the 80 lipid molecules were significantly altered in border and aneurysm areas, respectively, compared with those in normal areas of TAAA. Ten phosphatidylcholines (PCs), including PC(34:2) (*p* = 0.002) and PC(36:3)a (*p* = 0.009), and eight SMs, including SM(34:0) (*p* = 0.021) and SM(42:2)b (*p* = 0.001), were increased in the aneurysm area. In addition, six cholesterols (Chs)/ChEs and all triacylglycerols (TGs), except for TG(50:0)b, also increased. In contrast, two ether-type phosphatidylethanolamines (ePEs), i.e., PE(38:5e)b (*p* = 0.021 for border and *p* < 0.001 for aneurysm) and PE(40:7e) (*p* = 0.007 for border and *p* < 0.001 for aneurysm), gradually decreased in the border and aneurysm areas of TAAA. In addition, total quantified ePE (*p* = 0.050 for border and *p* = 0.001 for aneurysm) also gradually decreased in the border and aneurysm areas. Compositional determination of the fatty acid side chains demonstrated that decreased ePEs were all plasmalogens; PE(38:5e)b was PE(18:0p/20:4) and PE(40:7e) was PE(20:2p/20:4) (Fig. 3d). In contrast to the findings in TAAA, no lipid molecules, except for PC(36:5e) and G1Cer(42:1), showed significant alterations among normal, border, and aneurysm areas of TNAA.

### Comparison of oxylipins among normal, border, and aneurysm areas in TAAA and TNAA

**Figure 4:**
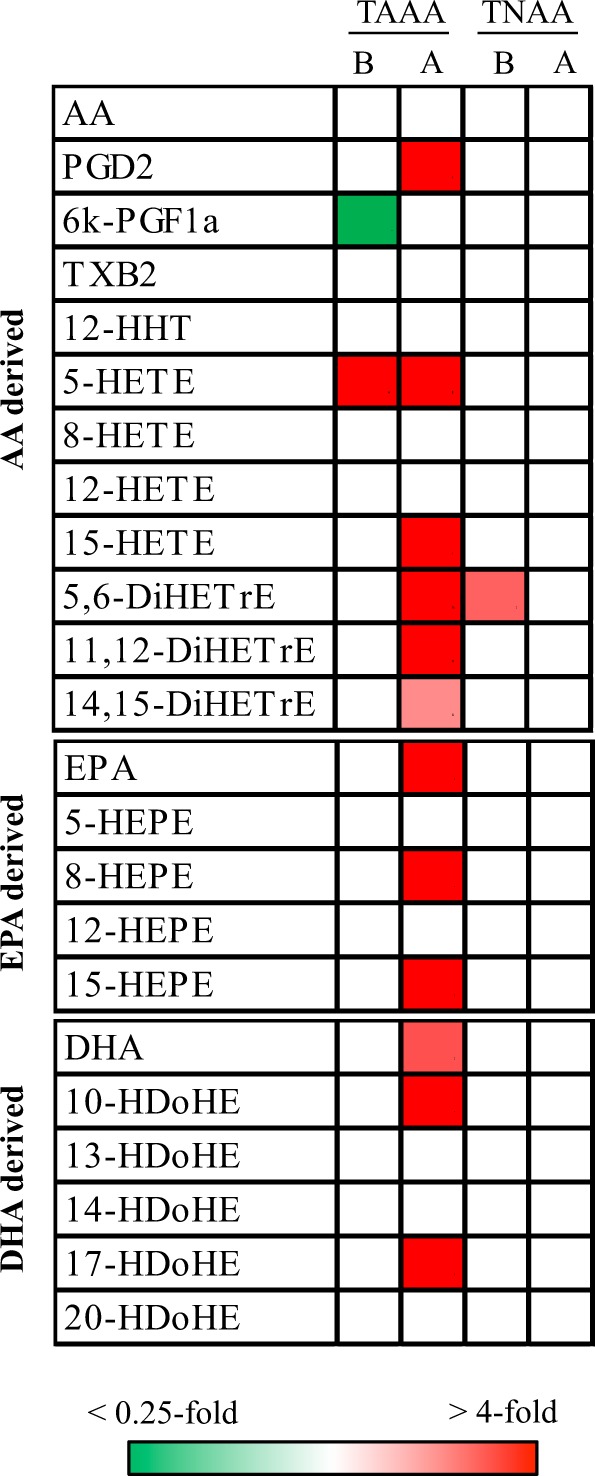
Heatmaps for molecules identified by targeted oxylipin analysis of aortic media among areas of TAAA and TNAA. Heatmaps with significantly different (*p* < 0.05) lipid molecules were generated using mean fold changes in the levels of individual molecules calculated as ratios of each area to the normal area. Vacant white cells indicate molecules that were quantified, but their levels were not significantly different. B: border, A: aneurysm, AA: arachidonic acid, PGD_2_: prostaglandin D_2_, 6k-PGF_1a_: 6-keto prostaglandin F_1a_, TXB_2_: thromboxane B_2_, 12-HHT: 1_2_-hydroxyheptadecatrienoic acid, HETE: hydroxyeicosatetraenoic acid, DiHETrE: dihydroxyeicosatrienoic acid, EPA: eicosapentaenoic acid, HEPE: hydroxyeicosapentaenoic acid, DHA: docosahexaenoic acid, HDoHE: hydroxydocosahexaenoic acid.

Next, we compared targeted lipid profiles of oxylipins in the aortic media among normal, border, and aneurysm areas obtained from patients with TAAA and TNAA (Fig. 4). Processing of targeted lipid molecules yielded 23 lipid molecules (Fig. 4 and Supplemental Tables S4 and S5). Eicosapentaenoic acid (EPA) and docosahexaenoic acid (DHA) but not arachidonic acid (AA) increased in aneurysm area of TAAA. In addition, several oxylipins, including PGD_2_ (*p* = 0.001) and 15-lipoxygenase (LOX) metabolites (15-hydroxyeicosatetraenoic acid [15-HETE] (*p* = 0.005), 15-hydroxyeicosapentaenoic acid [15-HEPE] (*p* = 0.020), and 17-hydroxydocosapentaenoic acid [17-HDoHE] (*p* = 0.020)), increased in the aneurysm area. In contrast to the findings in TAAA, none of the lipid molecules except for 5,6-dihydroxyeicosatetraenoic acid (DiHETrE) changed in the normal, border, and aneurysm areas of TNAA.

### Comparison of lipid profiles among normal, border, and aneurysm areas in AAAA

**Figure 5:**
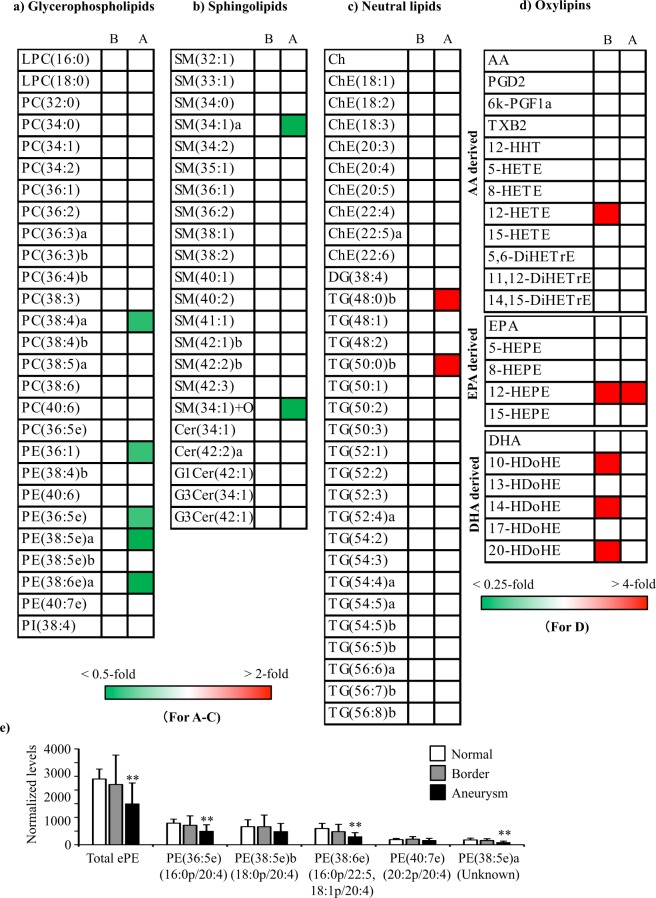
Heatmaps for molecules identified by lipidomic analysis of aortic media in normal, border and aneurysm areas of AAAA. (**a–d**) Heatmaps with significantly different (*p* < 0.05) lipid molecules were generated using mean fold changes in the levels of individual molecules calculated as ratios of each area to normal area. Vacant white cells indicate molecules that were quantified, but their levels were not significantly different. (**e**) Levels of ether-type PEs for the three areas from AAAA. Data are presented as summed normalised ion peak heights of each peak of the sample groups and shown as means ± SDs. *p < 0.05, **p < 0.01 in paired t-tests comparing border or aneurysm areas and normal areas. Combinations of fatty alcohols and fatty acids are shown underneath each molecule. The last lowercase letters (a,b) in lipid names represent molecules with the same carbon and double bond numbers, but have different fatty acid compositions (molecules with same m/z in MS with different retention time in LC). Abbreviations are described in Table 2 and Fig. 4.

We further compared lipid profiles of the aortic media among normal, border, and aneurysm areas obtained from patients with AAAA (Fig. 5). Despite the limited number of samples, several lipid molecules were significantly altered in the border and aneurysm areas when compared with those in the normal areas. Two TGs, i.e., TG(48:0)b (*p* = 0.043) and TG(50:0)b (*p* = 0.018), increased in the aneurysm area. In addition, total TGs also tended to increase, although the changes were not significant (Supplemental Fig. S2). Moreover, two 12-LOX metabolites, i.e., 12-HETE (*p* = 0.032) and 14-HDoHE (*p* = 0.029), increased in the border area, and another 12-LOX metabolite, i.e., 12-HEPE (*p* = 0.042 for border and *p* = 0.038 for aneurysm), increased in both the border and normal areas. Two other DHA metabolites, i.e., 10-HDoHE (*p* = 0.037) and 20-HDoHE (*p* = 0.048), were also increased in the border area. In contrast, three ePEs, i.e., PE(36:5e) (*p* = 0.004), PE(38:5e)a (*p* = 0.007), and PE(38:6e) (*p* = 0.002), SM(34:1)a (*p* = 0.034), and SM(34:1) + O (*p* = 0.009) decreased in the aneurysm area of AAAA. In addition, total measurable ePE (*p* = 0.004) also decreased in the aneurysm area (Fig. 5E). Compositional determination of the fatty acid side chains demonstrated that the two decreased ePEs were plasmalogens (PE(36:5e) was PE(16:0p/20:4) and PE(38:6e) was PE(16:0p/22:5, 18:1p/20:4)), whilst the composition of the other was undetermined. The decrease in total ePEs in the aneurysm area of AAAA was similar to that of TAAA, although the decreased ePE molecules were different between AAAA and TAAA.

### Comparison of lipid profiles in normal areas of TAAA and TNAA compared with those in CNTA

**Figure 6:**
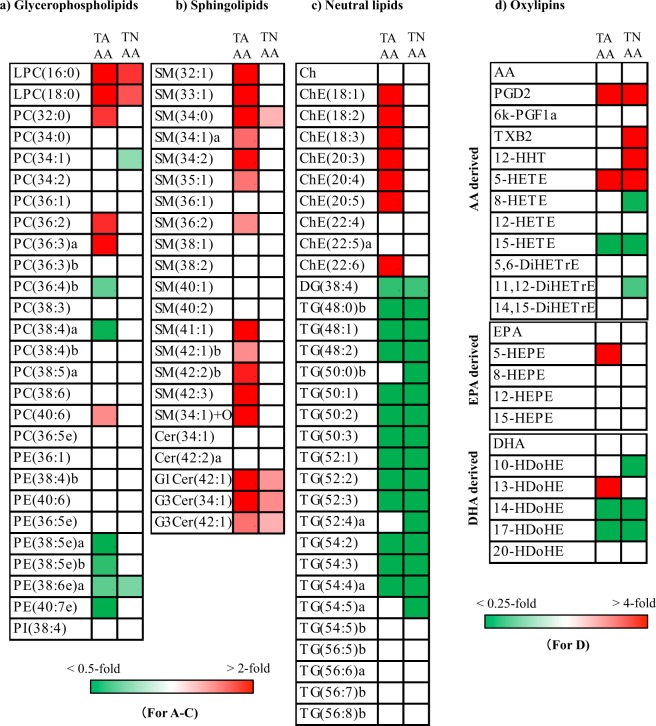
Heatmaps for molecules identified by lipidomic analysis of normal aortic media of TAAA and TNAA against CNTA (control thoracic aortic media of patients without vascular disease). Heatmaps with significantly different (*p* < 0.05) lipid molecules were generated using mean fold changes in the levels of individual molecules calculated as ratios of either TAAA or TNAA to CNTA. Vacant white cells indicate molecules that were measurable, but their levels were not significantly different. The last lowercase letters (a,b) in lipid names represent molecules with the same carbon and double bond numbers, but different fatty acid compositions (molecules with same *m/z* in MS with different retention times in LC). Other abbreviations are described in Table 2 and Fig. 4.

Next, we compared lipid profiles in normal areas of TAAA and TNAA with CNTA from patients without vascular disease (Fig. 6). Unlike differences between aneurysm/boarder areas and normal areas, the overall lipid profiles of the aortic media in TAAA and TNAA showed similar trends when compared with those in CNTAs. Lysophosphatidylcholines (LPCs), such as LPC(16:0) (*p* = 0.022 for TAAA and *p* = 0.041 for TNAA) and LPC(18:0) (*p* = 0.021 for TAAA and *p* = 0.039 for TNAA); glycosylceramides (GCers), such as G1Cer(42:1) (*p* = 0.020 for TAAA and *p* = 0.040 for TNAA) and G3Cer(34:1) (*p* = 0.018 for TAAA and *p* = 0.019 for TNAA); increased in both TAAA and TNAA. In addition, several oxylipins, such as PGD_2_ (*p* < 0.001 for TAAA and *p* = 0.016 for TNAA) and 5-LOX metabolites (5-HETE (*p* = 0.001 for TAAA and *p* = 0.048 for TNAA)), also increased in both conditions. Only SMs, such as SM(34:2) (*p* < 0.001) and SM(42:3) (*p* = 0.003), and ChEs, such as ChE(18:1) (*p* < 0.001) and ChE(18:2) (*p* < 0.001), increased specifically in TAAA. In contrast, ePEs, such as PE(38:6e)a (*p* = 0.008 for TAAA and *p* = 0.023 for TNAA), DG(38:4) (*p* = 0.011 for TAAA and *p* = 0.015 for TNAA), and TGs, such as TG(48:1) (*p* = 0.014 for TAAA and *p* = 0.009 for TNAA) and TG(52:2) (*p* = 0.013 for TAAA and *p* = 0.014 for TNAA), decreased in both TAAA and TNAA. In addition, 12-LOX and 15-LOX metabolites, i.e., 14-HDoHE (*p* = 0.031 for TAAA and *p* = 0.024 for TNAA), 17-HDoHE (*p* = 0.022 for TAAA and *p* = 0.022 for TNAA), and 15-HETE (*p* = 0.027 for TAAA and *p* = 0.029 for TNAA), also decreased in both TAAA and TNAA.

## Discussion

Alterations in lipid profiles emerged as a distinctive consequence of pathophysiological alterations in many diseases. In the present study, we first focused on alterations in lipid profiles in the aortic media from normal, border, and aneurysm tissues in patients with TAAA, TNAA, and AAAA, and then compared normal areas of aortic aneurysm patients with those from patients without vascular disease.

Changes in lipid profiles were evident in normal, border, and aneurysm areas in TAAA but not TNAA. Lipid profiles also changed among these areas of AAAA. Consistent with previous findings in atherosclerotic lesions^16,17^, our findings clearly demonstrated ChE + O accumulation in both TAAA and AAAA. In addition, we found several notable features of TAAA, including decreased ePE; increased PC, SM, Ch/ChE, and TG; and increased PGD_2_ and 15-LOX metabolites. Moreover, we also found decreased ePE and increased 12-LOX metabolites in AAAA, despite limited sample numbers.

Consistent with our findings, grade-associated increases in phospholipids, such as PCs and SMs, as well as Ch/ChEs and TGs in elastin from the aortic media have been characterised in atherosclerotic aortas^18,19^. In contrast, we found that grade-associated decreases in ePE were common features of both TAAA and AAAA, suggesting that decreased ePE in the aortic media could play a pivotal role in atherosclerotic aortic aneurysm development. In addition, our results demonstrated that decreased ePEs were all plasmalogens. Although the role of ePE in the aortic media is unclear, the major class of ePEs, i.e., plasmalogens, has been proposed to act as free radical scavengers^20^. Thus, ePE levels may have been reduced by oxidative stress generated during atherosclerotic events. Alternatively, alterations in ePE levels may be associated with cell transitions^21,22^. The mesenchymal-to-epithelial transition and mesenchymal-to-endothelial transition are associated with increased ePE levels. Conversely, the epithelial-to-mesenchymal transition is associated with decreased ePE levels. Because the mesenchymal transition acquires migratory and invasive properties, decreased ePE levels may be associated with increased migratory and invasive properties. In atherosclerotic plaques, macrophages transform into foam cells (lipid-laden macrophages), and vascular smooth muscle cells (VSMCs) proliferate and migrate from media to intima^23,24^. In addition, decreased VSMC contents in the aortic media, i.e., thinning of the aortic media, is a key feature in advanced-stage atherosclerotic aortic aneurysm development^6^. Thus, it is also possible that decreased ePE in the aortic media during atherosclerotic aortic aneurysm development reflects increased migratory and invasive properties of VSMCs and decreased VSMC contents in the aortic media. In a similar manner, increased levels of TGs and Ch/ChEs in the aortic media during atherosclerotic aortic aneurysm development may also be associated with the transition of VSMCs to migratory and invasive cell types.

Lipocalin-type PGD_2_ synthase (L-PGDS), which acts downstream of cyclooxygenase to produce PGD_2_, is elevated in atherosclerotic plaques^25^. Consistent with this previous report, our current study demonstrated that PGD_2_ levels were elevated in the aortic media during TAAA development. Moreover, knockout of L-PGDS accelerates aortic lipid accumulation and development of atherosclerosis in mice^26^. Thus, increased PGD_2_ in the aortic media may counteract the TAAA development, although the role of PGD_2_ in TAAA development remains unclear. Notably, L-PGDS expression levels are inversely correlated with matrix metalloproteinase 9 (MMP-9) levels, which are elevated to promote degradation of elastin and collagen matrices during the development of atherosclerotic aortic aneurysms^27^ and in atherosclerotic plaques^28^. In addition, L-PGDS inhibits the migration of VSMCs obtained from diabetic rats^29^. Therefore, suppression of both MMP-9 activity and VSMC migration may be one of the downstream effects of PGD_2_ in TAAA development.

In addition to PGD_2_ synthesis, synthesis of 5-LOX, 12-LOX, and 15-LOX metabolites has been reported to contribute to the development of atherosclerosis^30–32^. Thus, increased LOX metabolites also play important roles in the development of atherosclerotic aortic aneurysms. However, increased levels of 15-LOX metabolites (i.e., 15-HETE, 15-HEPE, and 17-HDoHE) during aortic aneurysm development were only observed in TAAA, whereas increased levels of 12-LOX metabolites (i.e., 12-HETE, 12-HEPE, and 14-HDoHE) during aortic aneurysm development were only observed in AAAA. Although the reason for this discordant upregulation of LOX metabolite levels between TAAA and AAAA remains to be elucidated; however, we hypothesise that these differences may be related to the different cell origins of VSMCs in the thoracic and abdominal aorta^33^. In addition, the different levels of LOX metabolites may contribute to differences in extracellular matrix density and responses of VSMCs to vasoactive growth factors^34^. The heterogeneity of VSMC origins may also explain the diverse production of LOX metabolites in the media of TAAA and AAAA.

Along with alterations in the lipid profiles during aortic aneurysm development, we also demonstrated alterations in lipid profiles in normal areas of the aortic media in both TAAA and TNAA, when compared with CNTAs without vascular disease. These alterations were similar between TAAA and TNAA; therefore, these lipid alterations could represent risky transitional markers of the aortic media leading to thoracic aortic aneurysm, although we cannot rule out the possibility that these alterations may be attributed to age. During the development of thoracic aortic aneurysms, decreased ePE and increased PGD_2_ and 5-LOX metabolites were also observed. In addition, increased GCers and LPCs and decreased TGs and 12- and 15-LOX metabolites were noted in the aortic media of thoracic aortic aneurysms. Extracellular signal-regulated kinase (ERK) has been shown to mediate platelet-derived growth factor-directed migration of VSMCs^35^. Moreover, GCers and LPCs can activate ERK signalling in VSMCs^36,37^. Thus, increased GCers and LPCs may represent the enhanced migratory activity of VSMCs by activating ERK signalling, which indicates the status of VSMCs in the aortic media leading to thoracic aortic aneurysm development. In the normal aortic media prior to thoracic aortic aneurysm development, 5-LOX metabolites increased, but 12- and 15-LOX metabolites decreased. Although the pathological implications of these lipid alterations have not been clarified, 5-HETE and 12- HETE/15-HETE have been shown to have opposite effects on insulin secretion in isolated pancreatic islets^38^. Thus, 12- and 15-HETE may also counteract 5-HETE in the initiation and development of thoracic aortic aneurysms, and imbalances may enhance the risk of initiation of thoracic aortic aneurysms.

There were several limitations in the present study. First, this study was performed with a limited number of patients; i.e., the patient numbers of TAAA, TNAA, AAAA and CNTA are 30, 19, 11 and 8, respectively. Especially, those of AAAA and CNTA are limited; therefore, only the comparisons of each lipid for TAAA provided false discovery rate (FDR) scores of less than 0.05. Second, there was a large age gap between patients with TAAA or AAAA and those with CNTA. This is mainly because of age limitation to those who can receive heart transplantation in Japan. We have once collected aortas from autopsy patients who had not been diagnosed as vascular diseases in the age range comparable to or more than TAAA and AAAA patients; their aortic tissues were all shown to have atherosclerotic denaturation by pathological examinations and not usable as normal control. There was also age difference between patients with TAAA or AAAA and those with TNAA. Third, there was personal heterogeneity of collected aortic tissue, and three areas (normal, border, and aneurysm) were not uniformly dissected and collected from aortic aneurysm tissues. Last, we performed lipidomic analysis of aortic media of aortic aneurysm, and other aortic wall components, such as intima and adventitia, and endothelial cells, have not been analysed. Therefore, we need to interpret and discuss the data under these limitations.

In conclusion, our comprehensive lipidomics approach using the aortic media of TAAA, TNAA, and AAAA revealed changes in lipid profiles in aortic aneurysm development for the first time. Additionally, from analyses of lipid profiles of the aortic media from TAAA and TNAA against CNTAs, we demonstrated lipid alterations in the thoracic aortic media that may initiate phenotype changes in VSMCs, leading to the development of aortic aneurysms. Through elucidation of the molecular mechanisms of these changes in lipidomics, the pathophysiology of atherosclerotic aortic aneurysms could be understood in greater detail, leading to the development of novel drugs to prevent and treat atherosclerotic aortic aneurysms.

## Methods

### Patients and tissue samples

This study followed the principles of the Declaration of Helsinki, and all patients provided written informed consent for tissue sampling and omics analyses, including lipidomics analyses. The protocol was approved by the Institutional Review Board of National Cerebral and Cardiovascular Center (Approval No. M22-029) and National Institute of Health Sciences (Approval No. 195-3), Japan. Aortic wall tissues were collected from patients undergoing elective surgery for aortic aneurysm, defined as TAAA (N = 30), TNAA (N = 19, such as connective tissue diseases, including Marfan syndrome), and AAAA (N = 11). CNTAs (N = 8) were collected from the root portions of aortas accompanied with hearts excised from patients who received heart transplantation and had no prominent symptoms of atherosclerosis and vascular disease. Details of patient characteristics are listed in Table 1. Statistical differences in age, body mass index, aneurysm size, and biochemical test data were determined by Kruskal-Wallis tests followed by Dunn’s multiple comparison tests, using GraphPad Prism 6 (GraphPad Software, San Diego, CA, USA).

Aortic walls were divided into three areas according to the degree of progression from the viewpoint of surgeons: aneurysm area, maximum diameter region of the aorta; border area, intermediate region between the aneurysm and normal areas; normal area, normal diameter and shape region of the aorta. Each tissue was further dissected into three layers, i.e., the intima, media, and adventitia. Aortic media used for lipidomic analysis were divided into pieces, weighed, and frozen in liquid nitrogen. Aortic tissues containing dissociations or calcifications were excluded to avoid problems associated with tissue heterogeneity. As controls, aortic media of root portions of aortas from recipient hearts were prepared using the same procedures. All samples were stored at −80 °C until lipidomic analysis.

### Histology

Portions of aortic tissues for pathological examinations and histological analyses (approximately 1 × 1 cm) were firstly separated, fixed overnight with 4% formalin (neutral pH), and embedded in paraffin without dissection into three layers. Sections (3 µm thick) were acquired using a sledge microtome, mounted on slides, and stained with hematoxylin and eosin, elastica van Gieson, and Masson’s trichrome to determine aortic wall morphology. CNTAs from patients without vascular disease were processed according to the same protocol.

### Lipid extraction

Lipid extraction from tissues was performed as described previously^39^. Briefly, lipids were extracted from 9 or 10 mg tissue by Bligh and Dyer’s method with a few modifications. The lower phase (corresponding to 20 µg of tissue) was used for measurement of phospholipids, sphingolipids, neutral lipids, and acylcarnitines. The upper phase (corresponding to 3 mg of tissue) was subjected to solid extraction and was used for measurement of oxylipins and their parental substrate polyunsaturated fatty acids (PUFAs). PC(16:0/16:0)-d_6_ (Larodan Fine Chemicals, Malmo, Sweden), TG(16:0/16:0/16:0)-^13^C_3_ (Larodan Fine Chemicals), and leukotriene B_4_-d_4_ (LTB_4_-d_4_; Cayman Chemical, Ann Arbor, MI, USA) were added as internal standards before extraction.

### Nontargeted detection and measurement of phospholipids, sphingolipids, and neutral lipids

Phospholipids, sphingolipids, and neutral lipids were quantified using LC/time-of-flight MS (TOFMS; ACQUITY UPLC System [Waters, Milford, MA, USA]/LCT Premier XE [Waters]), as described previously^40^. Aortic samples of normal, border, and aneurysm areas were randomised across the run. Because of limitations in sample numbers within each run sequence, samples were divided into four series of run sequences. Raw data obtained by LC/TOFMS were processed using the 2DICAL software (Mitsui Knowledge Industry, Tokyo, Japan), which allowed detection and alignment of the ion peaks of each ionised biomolecule obtained at the specific *m/z* and column retention time (RT). The main parameters of 2DICAL were set as described previously, with a few modifications^40^. To extract the ion peaks of phospholipids (LPC, PC, ether-type PC, phosphatidylethanolamine, ePE, PI, phosphatidylglycerol/bismonophosphotidate) and sphingolipids (SMs, ceramide, GCer), the RT range was from 2.0 to 38.0 min in the negative ion mode; whereas for ion peaks of neutral lipids (DG, Ch/ChE, coenzyme Q, TG, ether-type TG), the RT range was from 2.0 to 60.0 min in the positive ion mode. Extracted ion peaks were subjected to identification of lipid molecules by comparison of the ion features, including RT, *m/z*, preferred adducts, and in-source fragments, of the experimental samples with those of our reference library of lipid molecule entries, as described previously^40^. Structural analysis of fatty acid side chains from selected ePEs was performed by LC/Fourier transform MS (LTQ Orbitrap XL; Thermo Fisher Scientific, Waltham, MA, USA) as previously described^40^.

### Targeted measurement of PUFAs and their metabolites

Oxylipins and PUFAs were quantified by a targeted approach using LC/MS-MS (ACQUITY UPLC System/5500QTRAP quadrupole-linear ion trap hybrid mass spectrometer; AB Sciex, Framingham, MA, USA), as described previously^40^. The aortic samples were divided in the same manner as for nontargeted measurement and subjected to analyses. Targeted lipid molecules were annotated by comparison of RT, parent ions, and MS/MS ion fragments with standard lipid molecules using MultiQuant Software (Version 2.1; AB Sciex).

### Nontargeted lipidomics data processing

The data cut off point for nontargeted measurements was set at 50 for negative ion mode and 100 for positive ion mode in height. Using our non-targeted lipidomics platform, the majority of ion peaks lower than these cut off values had vague peak shapes and/or were interrupted by noise signals and were therefore not suitable for quantitative analysis. Due to the limited number of subjects, this study spanned multiple run sequences and therefore required the variation resulting from instrument inter-run tuning differences to be corrected for quantitative analysis. In addition, due to limited tissue availability and heterogeneity, we were unable to use quality control samples or pooled samples. Therefore, we used the median value of each lipid from the aortic TAAA samples, which were the most abundant and were included in all run sequences, to normalise multiple run sequences for the levels of each lipid in every sample of each run sequence. In addition, we set measurable limits such that 50% of samples within each run sequence exceeded the data cut off point. Moreover, to correct variations in each sample run, the intensity of each extracted ion peak was normalised to that of the major adduct ion of internal standard (PC[16:0/16:0]-d_6_ + HCOO^−^ for phospholipids and sphingolipids, and TG[16:0/16:0/16:0]-^13^C_3_ + NH_4_^+^ for neutral lipids). The relative standard deviation of the internal standards (PC[16:0/16:0]-d_6_, and TG[16:0/16:0/16:0]-^13^C_3_), which were used to monitor experimental quality during extraction, measurement, and data processing, were 9.7%, and 15.5%, respectively. For samples with missing values for a lipid, the minimum relative level of each lipid in all measurable samples was used instead. All data obtained by nontargeted measurements were presented as normalised levels of ion peak intensity. Comparisons of the metabolite levels were performed by t-test analyses to assess statistical differences and FDRs were calculated using the Benjamini-Hochberg method. Due to limited sample numbers, in this study, *p* values of less than 0.05 without FDR consideration were regarded as showing statistical significance for lipidomic measurements. The processed data, average values, and standard deviations of the levels of lipid molecules as well as statistical values (*p* value and FDR) are presented in Supplemental Tables S2 and S3.

### Targeted lipidomics data processing

The data cut off point for targeted measurements was set at a signal-to-noise ratio of 10. For samples with missing values for a metabolite, the minimum observed value of the metabolite among all samples was applied in targeted measurements. Data processing for quantitative analysis was conducted in the same manner as for the nontargeted lipidomics, except that the area of each ion peak from targeted lipid molecules was normalised to that of the major adduct ion the internal standard (LTB_4_-d_4_-H^+^) to correct for variation in each sample run. The relative standard deviation of the internal standards (LTB_4_-d_4_), which were used to monitor experimental quality during extraction, measurement, and data processing, were 15.2%. For samples with missing values for a lipid, the minimum relative level for each lipid in all measurable samples was used instead. All data obtained by targeted measurements were presented as normalised values against the median values of all quantified samples. Comparisons of the metabolite levels were performed by t-test analyses to assess statistical differences and FDR were calculated using the Benjamini-Hochberg method. Due to limited sample numbers, in this study, *p* values of less than 0.05 without FDR consideration were regarded as showing statistical significance for lipidomic measurements. The processed data, average values, and standard deviations of the levels of lipid molecules as well as statistical values (*p* value and FDR) are presented in Supplemental Tables S4 and S5.

## Supplementary information

Supplementary Figures

Supplemental Tables

## References

[1] Sakalihasan N, Limet R, Defawe OD (2005). Abdominal aortic aneurysm. Lancet..

[2] Golledge J, Eagle KA (2008). Acute aortic dissection. Lancet..

[3] Elefteriades JA (2008). Thoracic aortic aneurysm: reading the enemy’s playbook. Curr. Probl. Cardiol..

[4] Golledge J, Muller J, Daugherty A, Norman P (2006). Abdominal aortic aneurysm: pathogenesis and implications for management. Arterioscler. Thromb. Vasc. Biol..

[5] Shimizu K, Mitchell RN, Libby P (2006). Inflammation and cellular immune responses in abdominal aortic aneurysms. Arterioscler. Thromb. Vasc. Biol..

[6] López-Candales A (1997). Decreased vascular smooth muscle cell density in medial degeneration of human abdominal aortic aneurysms. Am. J. Pathol..

[7] Allaire E (2009). New insight in aetiopathogenesis of aortic diseases. Eur. J. Vasc. Endovasc. Surg..

[8] Ogita T (1997). Lysophosphatidylcholine transduces Ca2+ signaling via the platelet-activating factor receptor in macrophages. Am. J. Physiol..

[9] Fadok VA (2000). A receptor for phosphatidylserine-specific clearance of apoptotic cells. Nature..

[10] Ichimura Y (2000). A ubiquitin-like system mediates protein lipidation. Nature..

[11] Tapiero H, Ba GN, Couvreur P, Tew KD (2002). Polyunsaturated fatty acids (PUFA) and eicosanoids in human health and pathologies. Biomed. Pharmacother..

[12] Houjou T, Yamatani K, Imagawa M, Shimizu T, Taguchi R (2005). A shotgun tandem mass spectrometric analysis of phospholipids with normal-phase and/or reverse-phase liquid chromatography/electrospray ionization mass spectrometry. Rapid Commun. Mass Spectrom..

[13] Han X, Gross RW (2005). Shotgun lipidomics: electrospray ionization mass spectrometric analysis and quantitation of cellular lipidomes directly from crude extracts of biological samples. Mass Spectrom. Rev..

[14] Saito K (2016). Lipidomic Signatures and Associated Transcriptomic Profiles of Clear Cell Renal Cell Carcinoma. Sci Rep..

[15] Alecu I, Bennett SAL (2019). Dysregulated Lipid Metabolism and its Role in α-Synucleinopathy in Parkinson’s Diseas. e. Front Neurosci..

[16] Tertov VV, Kaplun VV, Mikhailova IA, Suprun IV, Orekhov AN (2001). The content of lipoperoxidation products in normal and atherosclerotic human aorta. Mol. Cell Biochem..

[17] Upston JM (2002). Disease stage-dependent accumulation of lipid and protein oxidation products in human atherosclerosis. Am. J. Pathol..

[18] Kramsch D (1969). The protein and lipid composition of arterial elastin in ageing and atherosclerosis. Exp Gerontol..

[19] Kramsch DM, Franzblau C, Hollander W (1971). The protein and lipid composition of arterial elastin and its relationship to lipid accumulation in the atherosclerotic plaque. J. Clin. Invest..

[20] Broniec A (2011). Interactions of plasmalogens and their diacyl analogs with singlet oxygen in selected model systems. Free Radic. Biol. Med..

[21] Sampaio JL (2011). Membrane lipidome of an epithelial cell line. Proc. Natl. Acad. Sci. USA.

[22] Furihata T (2015). Hydrocortisone enhances the barrier properties of HBMEC/ciβ, a brain microvascular endothelial cell line, through mesenchymal-to-endothelial transition-like effects. Fluids Barriers CNS..

[23] Glukhova MA (1987). Identification of smooth muscle-derived foam cells in the atherosclerotic plaque of human aorta with monoclonal antibody IIG10. Tissue Cell..

[24] Vollmer E (1991). Immunohistochemical double labeling of macrophages, smooth muscle cells, and apolipoprotein E in the atherosclerotic plaque. Pathol. Res. Pract..

[25] Eguchi Y (1997). Expression of lipocalin-type prostaglandin D synthase (beta-trace) in human heart and its accumulation in the coronary circulation of angina patients. Proc. Natl. Acad. Sci. USA.

[26] Ragolia L (2005). Accelerated glucose intolerance, nephropathy, and atherosclerosis in prostaglandin D2 synthase knock-out mice. J. Biol. Chem..

[27] Ishii T, Asuwa N (2000). Collagen and elastin degradation by matrix metalloproteinases and tissue inhibitors of matrix metalloproteinase in aortic dissection. Hum. Pathol..

[28] Cipollone F (2000). Balance between PGD synthase and PGE synthase is a major determinant of atherosclerotic plaque instability in humans. Arterioscler. Thromb. Vasc. Biol..

[29] Ragolia L, Palaia T, Koutrouby TB, Maesaka JK (2004). Inhibition of cell cycle progression and migration of vascular smooth muscle cells by prostaglandin D2 synthase: resistance in diabetic Goto-Kakizaki rats. Am. J. Physiol. Cell. Physiol..

[30] Mehrabian M (2002). Identification of 5-lipoxygenase as a major gene contributing to atherosclerosis susceptibility in mice. Circ. Res..

[31] Yamamoto S (1992). Mammalian lipoxygenases: molecular structures and functions. Biochim. Biophys. Acta..

[32] George J (2001). 12/15-Lipoxygenase gene disruption attenuates atherogenesis in LDL receptor-deficient mice. Circulation..

[33] Wolinsky H (1970). Comparison of medial growth of human thoracic and abdominal aortas. Circ. Res..

[34] Halloran BG, Davis VA, McManus BM, Lynch TG, Baxter BT (1995). Localization of aortic disease is associated with intrinsic differences in aortic structure. J. Surg. Res..

[35] Graf K (1997). Mitogen-activated protein kinase activation is involved in platelet-derived growth factor-directed migration by vascular smooth muscle cells. Hypertension..

[36] Bhunia AK, Han H, Snowden A, Chatterjee S (1996). Lactosylceramide stimulates Ras-GTP loading, kinases (MEK, Raf), p44 mitogen-activated protein kinase, and c-fos expression in human aortic smooth muscle cells. J. Biol. Chem..

[37] Yamakawa T (1998). Lysophosphatidylcholine stimulates MAP kinase activity in rat vascular smooth muscle cells. Hypertension..

[38] Yamamoto S, Ishii M, Nakadate T, Nakaki T, Kato R (1983). Modulation of insulin secretion by lipoxygenase products of arachidonic acid. Relation to lipoxygenase activity of pancreatic islets. J. Biol. Chem..

[39] Tajima Y (2013). Lipidomic analysis of brain tissues and plasma in a mouse model expressing mutated human amyloid precursor protein/tau for Alzheimer’s disease. Lipids Health Dis..

[40] Ishikawa M (2014). Plasma and serum lipidomics of healthy white adults shows characteristic profiles by subjects' gender and age. PLoS One..

