# Supplementary material for: Lipidomic signatures of aortic media from patients with atherosclerotic and nonatherosclerotic aneurysms
Source: Sci Rep. 2019 Oct 29;9:15472. doi: 10.1038/s41598-019-51885-4 (PMC6820727; doi:10.1038/s41598-019-51885-4)
Supplement: Supplementary file 1 — Supplementary Figures [file 41598_2019_51885_MOESM1_ESM.pdf]

Lipidomic signatures of aortic media from patients with atherosclerotic and nonatherosclerotic aneurysms

Kosuke Saito[1]*, Hiroaki Yagi[2]*, Keiko Maekawa[1], Mitsuhiro Nishigori[2], Masaki Ishikawa[1], Sayaka Muto[3], Tsukasa Osaki[2], Yutaka Iba[4], Kenji Minatoya[4], Yoshihiko Ikeda[3], Hatsue Ishibashi-Ueda[3], Hitoshi Ogino[4], Hiroaki Sasaki[4], Hitoshi Matsuda[4], Yoshiro Saito[1]†, Naoto Minamino[2]†

Supplementary Figures

Supplemental Fig. 1

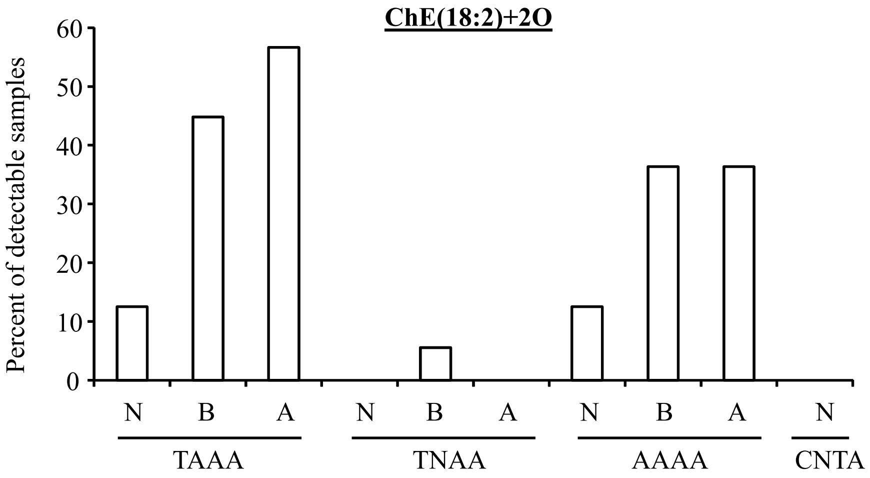

Percentage of detectable samples for a oxidized cholesterol (ChE[18:2]+2O) within the three areas of thoracic atherosclerotic aortic aneurysms (TAAA), thoracic nonatherosclerotic aortic aneurysms (TNAA), abdominal atherosclerotic aortic aneurysms (AAAA), and control aorta (CNTA). Data are presented as percentage of samples. N; normal area, B; border area, A; aneurysm area.

Supplemental Fig. 2

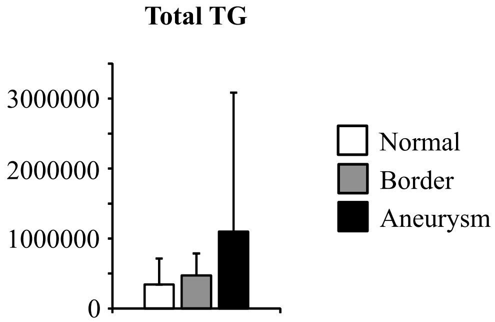

Levels of total triacylglycerol (TG) of the three areas from abdominal atherosclerotic aortic aneurysms (AAAA). Data are presented as normalized ion peak heights of each area of samples and shown as mean ± SD.
